# Supplementary material for: From sequence to enzyme mechanism using multi-label machine learning
Source: BMC Bioinformatics. 2014 May 19;15:150. doi: 10.1186/1471-2105-15-150 (PMC4229970; doi:10.1186/1471-2105-15-150)
Supplement: Additional file 2 — Java code of ml2db. Additional file ml2db_code.tar.gz contains the Java source code to run the multi-label machine learning experiments and save the results to database. The code’s Javadoc is included. [file 1471-2105-15-150-S2.zip › additional file 2/ml2db/ml2db/doc/main/ml2db/weka/ArffPropsFromFile.html]

ArffPropsFromFile


JavaScript is disabled on your browser.


- Overview
- Package
- Class
- Use
- Tree
- Deprecated
- Index
- Help

- Prev Class
- Next Class

- Frames
- No Frames

- All Classes

- Summary:
- Nested |
- Field |
- Constr |
- Method

- Detail:
- Field |
- Constr |
- Method


main.ml2db.weka

## Class ArffPropsFromFile

- java.lang.Object
- - uk.ac.standrews.utils.main.Initialised
  - - main.ml2db.weka.ArffProps
    - - main.ml2db.weka.ArffPropsFromFile

- All Implemented Interfaces:
  :   uk.ac.standrews.utils.main.Initialisable

  Direct Known Subclasses:
  :   MulanArffProperties

  ---

    

  ```
  public class ArffPropsFromFile
  extends ArffProps
  ```

  Contains the properties for a Weka ARFF file to be generated from database
  queries.
  It contains: 1. the properties read from a properties (.props) file 2. the
  path of the properties file itself 3. the generated name for the arff file
  including a timestamp 4. (optional, to be filled by other classes) some
  statistics for the data (number of attributes, classes, instances.

  Version:
  :   24-Mar-2007

  Author:
  :   Luna De Ferrari ldf(at)st-andrews.ac.uk

- - ### Field Summary

    Fields

    | Modifier and Type | Field and Description |
    | --- | --- |
    | `static java.lang.String` | `DATA_PROPERTIES_FILE_PATH_PROP` the path to the properties file itself, from which the other properties were read |
    | `static java.lang.String` | `INSTANCE_ATTRIBUTE_QUERY_PROP` The query to get the attributes for each instance |
    | `static java.lang.String` | `INSTANCE_CLASS_QUERY_PROP` The query to get the class value for each instance |
    | `static java.lang.String` | `TIMESTAMP_FIELD` the timestamp field |

    - ### Fields inherited from class main.ml2db.weka.ArffProps

      `ARFF_DATA_PROP, ARFF_FILE_PATH_PROP, CLASS_NAME_PROP, DATASET_NAME_PROP, DESCRIPTION_PROP, NUMBER_OF_ATTRIBUTES, NUMBER_OF_CLASS_VALUES, NUMBER_OF_CORE_PROPERTIES, NUMBER_OF_INSTANCES, NUMBER_OF_STATISTICS_PROPERTIES, XML_DATA_PROP, XML_FILE_PATH_PROP`
    - ### Fields inherited from class uk.ac.standrews.utils.main.Initialised

      `m_initialisationIsCorrect`
  - ### Constructor Summary

    Constructors

    Constructor and Description

    `ArffPropsFromFile(java.lang.String propertiesFilePath)`

    Constructor loads the properties from .props properties file
  - ### Method Summary

    Methods

    | Modifier and Type | Method and Description |
    | --- | --- |
    | `java.util.TreeSet<java.lang.String>` | `coreKeys()` The core ARFF properties, essential to generate a Weka ARFF file |
    | `boolean` | `corePropertiesAreCorrect(java.util.Properties props)` |
    | `int` | `getNumberOfCoreProperties()` The core ARFF properties, essential to generate a Weka ARFF file |
    | `java.lang.String` | `getPropertiesFilePath()` Get the properties file path |

    - ### Methods inherited from class main.ml2db.weka.ArffProps

      `checkPropertiesKeys, deriveXmlPath, getArffFilePath, getDbConn, getNumberOfStatsProperties, getProperties, getProperty, getXmlFilePath, propertiesAreCorrect, propertiesKeys, saveStatisticsAsProperties, statisticsKeys, statsPropertiesAreCorrect`
    - ### Methods inherited from class uk.ac.standrews.utils.main.Initialised

      `initialisationIsCorrect, setInitialisation, setInitialisationIsCorrect, setInitialisationNotCorrect`
    - ### Methods inherited from class java.lang.Object

      `equals, getClass, hashCode, notify, notifyAll, toString, wait, wait, wait`

- - ### Field Detail


    - #### DATA\_PROPERTIES\_FILE\_PATH\_PROP

      ```
      public static final java.lang.String DATA_PROPERTIES_FILE_PATH_PROP
      ```

      the path to the properties file itself, from which the other properties
      were read

      See Also:
      :   Constant Field Values


    - #### INSTANCE\_ATTRIBUTE\_QUERY\_PROP

      ```
      public static final java.lang.String INSTANCE_ATTRIBUTE_QUERY_PROP
      ```

      The query to get the attributes for each instance

      See Also:
      :   Constant Field Values


    - #### INSTANCE\_CLASS\_QUERY\_PROP

      ```
      public static final java.lang.String INSTANCE_CLASS_QUERY_PROP
      ```

      The query to get the class value for each instance

      See Also:
      :   Constant Field Values


    - #### TIMESTAMP\_FIELD

      ```
      public static final java.lang.String TIMESTAMP_FIELD
      ```

      the timestamp field

      See Also:
      :   Constant Field Values
  - ### Constructor Detail


    - #### ArffPropsFromFile

      ```
      public ArffPropsFromFile(java.lang.String propertiesFilePath)
      ```

      Constructor loads the properties from .props properties file

      Parameters:
      :   `propertiesFilePath` - the path to the properties file e.g.
          /home/me/myexperiment.props
  - ### Method Detail


    - #### coreKeys

      ```
      public java.util.TreeSet<java.lang.String> coreKeys()
      ```

      The core ARFF properties, essential to generate a Weka ARFF file

      **Overrides:**
      :   `coreKeys` in class `ArffProps`

      Returns:
      :   the essential properties to generate an arff file


    - #### corePropertiesAreCorrect

      ```
      public boolean corePropertiesAreCorrect(java.util.Properties props)
      ```

      **Overrides:**
      :   `corePropertiesAreCorrect` in class `ArffProps`


    - #### getNumberOfCoreProperties

      ```
      public int getNumberOfCoreProperties()
      ```

      The core ARFF properties, essential to generate a Weka ARFF file

      **Overrides:**
      :   `getNumberOfCoreProperties` in class `ArffProps`

      Returns:
      :   the essential properties to generate an arff file
          public TreeSet coreKeys() { TreeSet set = new
          TreeSet();
          set.add(ArffRecordFromPropsFile.ARFF\_FILE\_PATH\_PROP);
          set.add(ArffRecordFromPropsFile.CLASS\_NAME\_PROP);
          set.add(ArffRecordFromPropsFile.DATA\_PROPERTIES\_FILE\_PATH\_PROP);
          set.add(ArffRecordFromPropsFile.DATASET\_NAME\_PROP);
          set.add(ArffRecordFromPropsFile.DESCRIPTION\_PROP);
          set.add(ArffRecordFromPropsFile.INSTANCE\_ATTRIBUTE\_QUERY\_PROP);
          set.add(ArffRecordFromPropsFile.INSTANCE\_CLASS\_QUERY\_PROP);
          return set; }


    - #### getPropertiesFilePath

      ```
      public java.lang.String getPropertiesFilePath()
      ```

      Get the properties file path


- Overview
- Package
- Class
- Use
- Tree
- Deprecated
- Index
- Help

- Prev Class
- Next Class

- Frames
- No Frames

- All Classes

- Summary:
- Nested |
- Field |
- Constr |
- Method

- Detail:
- Field |
- Constr |
- Method
